# Analytical setup margin for spinal stereotactic body radiotherapy based on measured errors

**DOI:** 10.1186/s13014-021-01956-6

**Published:** 2021-12-07

**Authors:** Audrey Copeland, Addie Barron, Jonas Fontenot

**Affiliations:** 1 Department of Physics and Astronomy, Louisiana State University and Agricultural and Mechanical College, Baton Rouge, LA USA; 2 Department of Physics, Mary Bird Perkins Cancer Center, Baton Rouge, LA USA

**Keywords:** Spine, SBRT, Stereotactic, PTV, Margin

## Abstract

**Background:**

No consensus currently exists about the correct margin size to use for spinal SBRT. Margins have been proposed to account for various errors individually, but not with all errors combined to result in a single margin value. The purpose of this work was to determine a setup margin for five-fraction spinal SBRT based on known errors during radiotherapy to achieve at least 90% coverage of the clinical target volume with the prescription dose for at least 90% of patients and not exceed a 30 Gy point dose or 23 Gy to 10% of the spinal cord subvolume.

**Methods:**

The random and systematic error components of intrafraction motion, residual setup error, and end-to-end system accuracy were measured. The patient’s surface displacement was measured to quantify intrafraction motion, the residual setup error was quantified by re-registering accepted daily cone beam computed tomography setup images, and the displacement between measured and planned dose profiles in a phantom quantified the end-to-end system accuracy. These errors and parameters were used to identify the minimum acceptable margin size. The margin recommendation was validated by assessing dose delivery across 140 simulated patient plans suffering from various random shifts representative of the measured errors.

**Results:**

The errors were quantified in three dimensions and the analytical margin generated was 2.4 mm. With this margin applied in the superior/inferior direction only, at least 90% of the CTV was covered with the prescription dose for 96% of the 140 patients simulated with minimal negative effect on the spinal cord dose levels.

**Conclusions:**

The findings of this work support that a 2.4 mm margin applied in the superior/inferior direction can achieve at least 90% coverage of the CTV for at least 90% of dual-arc volumetric modulated arc therapy spinal SBRT patients in the presence of errors when immobilized with vacuum bags.

## Background

Stereotactic body radiotherapy (SBRT) has been shown to improve outcomes for spinal metastases compared to conventional radiotherapy [1]. Because of the steep dose gradients associated with spine SBRT plans, the choice of setup margins is critical. Setup or PTV margins can account for uncertainties in delivery and patient setup to ensure appropriate target coverage by irradiating a slightly larger volume of tissue including the intended treatment volume. Unfortunately, established margin formulae, such as that described by Van Herk, useful for conventional fractionation schemes may not be applicable to SBRT [2]. Consequently, a common ad hoc practice for spine SBRT planning involves isotropic PTV setup margins of up to two millimeters when feasible, which is reduced when overlapping a critical structure such as the spinal cord [3]. Others have reported using no setup margin at all for spine SBRT treatment planning [3]. This reduces the amount of normal tissue potentially unnecessarily irradiated, but does not allow any room for error. Unfortunately, neither approach relies on quantification and analysis of actual setup errors, and such approaches cannot guarantee or even estimate the probability of CTV coverage.

A potential margin calculation algorithm for SBRT treatments was previously described by Herschtal et al. [4]. The so-called SDE2 (sharp dose edge) algorithm calculates the required setup margin using a geometric modeling approach that utilizes random and systematic setup error distributions provided by the user. Additional details of the algorithm are described elsewhere [4]. Both Chang et al. and Lyons et al. utilized the SDE2 algorithm to determine SBRT margins for spine and prostate but they did not include an important source of setup error in their analysis: end-to-end system accuracy [5, 6].

All known sources of uncertainty should be included when determining a safe margin for treatments requiring a high level of precision [2]. Finnigan and Chang identified the components of error necessary for a margin recipe to be residual setup error, end-to-end system accuracy, and intrafraction motion [5, 7]. The residual setup error is the remaining difference in position between the CTV just before treatment and the planned position of the CTV which was used to create the treatment plan. The end-to-end system accuracy consists of the measured geometric shift between the planned dose distribution and the deliverable dose distribution. Finnigan notes many individual mechanical sources of error, but these can be combined together with comprehensive end-to-end testing starting from simulation with a planning CT image and measuring the accuracy of the resulting dose delivery on the linear accelerator [7]. Intrafraction motion consists of any patient or target motion during a fraction while radiation is being delivered. Since the patient population, equipment, and procedures may differ between facilities, error components should ideally be measured by each institution [8, 9].

The purpose of this work was to utilize the SDE2 algorithm to determine the setup margin required for spinal SBRT to achieve at least 90% CTV coverage and limit the spinal cord to within the defined tolerance values for at least 90% of patients in the presence of measured geometrical uncertainties [5, 10, 11]. Two fractionation schemes were investigated: a five-fraction and a single-fraction course.

## Methods

**Fig. 1:**
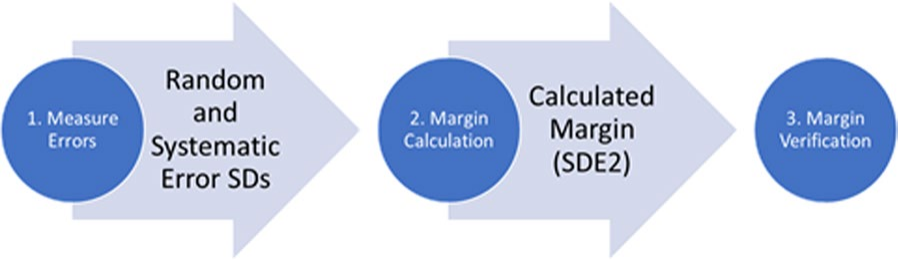
Workflow of this study

Uncertainties affecting the setup margin were measured, including residual setup error, intrafraction motion, end-to-end system accuracy, and penumbral width of the treatment plan. Figure 1 demonstrates the workflow used in this study, where measured errors and other necessary parameters were input into a margin calculation algorithm, and the generated margin was then validated. Since a spine SBRT program was not yet implemented at the time of this study, uncertainties were approximated from measurements of “similar” treatments (subsequently defined) at our institution. “Similar” treatments were identified based on similar treatment site, treatment modality, immobilization device type, and treatment time.

Residual setup error was estimated from pre-treatment cone-beam CT (CBCT) images of conventionally-fractionated treatments of spine and adjacent lesions. A total of 195 setup CBCT images for 20 patients were collected and analyzed to estimate the mean value with 95% confidence to within 0.2 mm, assuming the standard deviation of the data is similar to other reported values [5]. The residual setup error was measured retrospectively after the CBCT setup images were taken as a routine part of the patient setup procedure for the patients analyzed. Each case had CBCT setup images taken before delivery where the number of images for each patient ranged from 4 to 36. Each image was registered with the planning CT image using automatic registration software (XVI). The therapist then manually adjusted the registration if necessary, and the couch was automatically shifted. To measure the residual setup error, the couch shifts were added manually to the setup image and were assumed to be implemented perfectly by the couch. The simulation CT was re-registered with this shifted CBCT to measure the shift between the images. This small residual displacement is the amount of residual setup error for that patient for that fraction.

Intrafraction motion of spine SBRT patients was estimated using a commercial optical surface imaging system which recorded the translational and rotational displacement of the patient’s surface in real time (Catalyst HD, C-RAD GmbH, Berlin, Germany). Patients receiving surface imaging guidance for pelvic cancers were determined to be a suitable surrogate for spine patients. Breathing motion effects were minimized by selecting the pelvic region of the patient’s surface and by the averaging performed in the C-RAD software for the data recording. All patients either received dual-arc VMAT or 3D conformal therapy with conventional fractionation. These patients were immobilized with vacuum cushions (Vac-Lok™ Cushions, Civco Radiotherapy, Coralville, IA) from the knees down with a wingboard, which is similar to the planned immobilization of spine SBRT patients. The average time for treatment, defined as the time from first beam-on to the last beam-on, was 5 min and 5 s and was comparable to the approximately six-minute spine treatments. There were 2 spine and 24 pelvis patients who received between 4 and 23 fractions. A total of 409 sets of surface imaging data were collected for the 26 patients and analyzed to estimate the mean value with 95% confidence to within 0.2 mm. The average values and standard deviations of displacement in all directions were calculated over each fraction for each patient. Then, the patients were binned together and patient averages and standard deviations over all fractions were calculated. The root-mean-square sum of the patient standard deviations represents the random error and the standard deviation of the patient averages represents the systematic error [12].

The end-to-end system accuracy was previously measured, and the data was analyzed in this work to obtain the random and systematic error components [13]. The measurement procedure followed the process of a patient treatment through CT simulation and delivery on either Elekta Infinity with Agility Head and Versa HD linear accelerators (Elekta, Stockholm, Sweden) of a sample spine SBRT patient plan with CBCT image guidance to a commercial two-dimensional diode array (MapCHECK2 serial number: 76352038; Sun Nuclear Corporation, Melbourne, FL). In order to achieve 1 mm resolution, the diode array was set up on a motion stage, a platform which can be moved in more precise increments on the order of millimeters, on the treatment couch. The plan was delivered 10 times to the diode array and it was shifted by 1 mm using the motion stage between deliveries. Then, the dose profiles measured by the diode array were compared to the planned profiles obtained from the dose distribution in the treatment planning system in order to measure the shift between them.

The measure of end-to-end system accuracy is the distance between specified isodose points in the planned and measured dose profiles. This measurement procedure includes all sources of mechanical error causing a shift of the dose distribution from any of the systems involved from simulation to beam delivery, including the isocenter accuracies of the CBCT system and the radiation beam, the localization uncertainty of the CBCT system and the couch, and the MLC uncertainty [14]. A total of 14 dose planes were measured between both linear accelerators, where 10 planes were measured in the lateral direction, and 4 planes were measured in the longitudinal direction. Five profiles were analyzed through each plane where the diodes were spaced 1 cm apart. To represent the systematic error component, the standard deviation over the average planar shift values was calculated. The random component consists of the RMS sum of the individual intra-planar standard deviations [14]. Because there were no significant differences in the standard deviations of the measurements of the two directions, all measured planes were binned together. The standard deviations of the system accuracy were found individually for each linear accelerator used and there was not a significant difference between the machines, and so these were binned together as well.

**Fig. 2:**
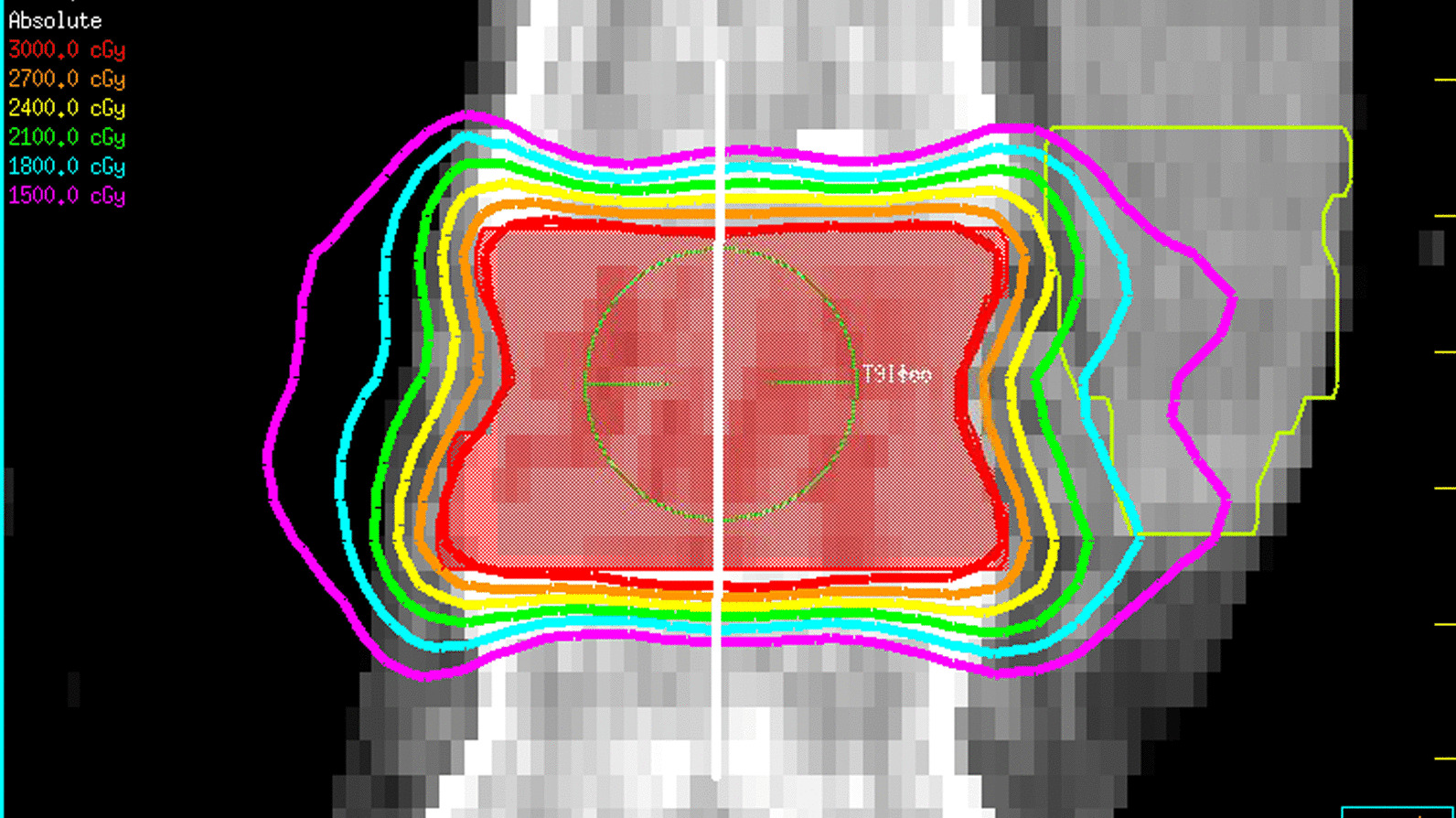
Coronal view of isodose lines resulting from the spinal SBRT plan

Since the setup margin value depends inversely on the penumbral width and increases with decreasing penumbra, the minimum penumbral width was measured on an actual five-fraction spinal SBRT plan used at MBPCC. The minimum penumbra, or sharpest penumbra, exists in the superior/inferior direction in a VMAT plan as seen in Fig. 2 because the edges of the beam there are mostly defined by the MLCs and not by the attenuation of other parts of the arc beam through the patient and past the target. The white line shows the line along which the distance between isodose lines were measured. Applying the minimum penumbral width to the margin recipe will yield the most conservative margin in terms of CTV coverage because it will result in larger margin within certain bounds [15]. To quantify the minimum penumbral width in the spinal SBRT VMAT plan, the distance between the 90% and 50% isodose lines was measured in the superior/inferior direction in a line through isocenter. This was done in the sagittal and coronal planes on the simulation CT images in the Pinnacle TPS both above and below the target volume in the original plan for four total measurements.

Next, the SDE2 algorithm was implemented to determine a setup margin for an SBRT treatment which would achieve the desired clinical outcomes of CTV coverage and minimum cord dose. The SDE2 algorithm is a simulation-based algorithm for calculating a margin for hypofractionated treatments, including SBRT treatments [4]. This algorithm is separated into three separate steps in which (1) a dose-population histogram table is generated, (2) a margin is determined by interpolating between table entries, and (3) the margin value is validated with Monte-Carlo type simulations. This margin calculation included all known sources of uncertainty contributing to a geometrical shift from the planned dose distribution resulting in dosimetric changes to the target and surrounding normal tissue. The calculation algorithm requires user-specified inputs of fraction number, systematic error, random error, desired minimum volume of target receiving prescription dose, and desired minimum population percentage receiving at least that dose. The number of fractions was set to five and one. The minimum acceptable dose to a point in the CTV was set at 90% of the prescription dose, in accordance with recommended dose limits in RTOG 0631. The minimum proportion of the population to receive the prescription dose to at least this coverage level was also set to 90% as this has been used previously where margins have been calculated [5, 10, 11]. The random and systematic errors were calculated as the combination of each measured error type: end-to-end system accuracy, intrafraction motion, and residual setup error as in Eqs. 1 and 2. To achieve the most conservative margin which could be reasonably applied isotropically, the maximal directional errors were used for each error component. For example, for the random intrafraction error, the largest component was in the superior/inferior (Y) direction, so that value was used in Eq. 1.

1 $${\sigma }_{rand}=\sqrt{{\sigma }_{IF,rand}^{2}+{\sigma }_{SE,rand}^{2}+{\sigma }_{ETE,rand}^{2}}$$

2 $${\Sigma }_{\mathrm{sys}}=\sqrt{{\Sigma }_{\mathrm{IF},\mathrm{sys}}^{2}+{\Sigma }_{\mathrm{SE},\mathrm{sys}}^{2}+{\Sigma }_{\mathrm{ETE},\mathrm{sys}}^{2}}$$

The margins for five-fraction and single-fraction cases calculated from the SDE2 algorithm and from the measured parameters were validated by implementing random patient offsets from the measured error distributions and calculating the resulting dose to the CTV and the spinal cord in the TPS with each different margin applied [16, 17]. The cord tolerance was 23 Gy to 10% of its subvolume defined in the report of Task Group 101 and a 30 Gy maximum dose for the five-fraction case [18]. For the single-fraction case, the tolerances were 10 Gy to 10% of the subvolume and 14 Gy maximum dose. Four different margins were evaluated on CTV coverage and cord dose and compared for the five-fraction case. The results of adding the analytical margin both isotropically and in the superior/inferior direction only were compared with the standard 2 mm margin expansion typically used as well as with adding no margin to the CTV. Adding the analytical margin in the superior/inferior direction only was done because this is the direction with the minimum penumbral width which the margin calculation assumed. The other directions have much wider or larger penumbras and the calculated margin which used the minimum penumbral width is an overestimation of what is necessary to achieve the treatment objectives in the right/left and anterior/posterior directions.

New plans were created for the new PTVs resulting from each margin expansion, which were all based on one clinical spinal SBRT plan. The original plan was a dual-arc, VMAT, spinal SBRT plan which was treated in five fractions to a prescription dose of 3000 cGy at MBPCC. The coverage of the CTV with the prescription dose was 97% in the original plan. The maximum cord dose was 2147 cGy. The new plans were created by copying the original plan to a new trial and changing the inverse planning objectives to reflect the modified PTV. These plans were adjusted to meet the dosimetric values of the original plan but for the newly created PTVs. The goal was to create very similar plans to the original to achieve the fairest comparison between the different margins.

A script was written in the Pinnacle TPS to automatically perform the steps required for each margin validation, which are as follows: apply random shift to the isocenter simulating a patient setup error, recalculate the dose, then export desired DVH parameters related to the CTV and spinal cord. These values were the percentage of the CTV receiving 100% of the prescription dose, the maximum point dose in the spinal cord, and the volume of the spinal cord receiving the tolerance dose. A separate Matlab script was used to generate the necessary random shifts for each fraction in each direction. A total of 140 simulated treatments were performed for each margin case to estimate the percentage of treatments receiving at least 90% of the prescription dose to the CTV to within 10% uncertainty calculated from power analysis.

## Results

The standard deviations of random and systematic components of residual setup error, intrafraction motion, and end-to-end system accuracy are shown in Table 1. The minimum average penumbral width over the four superior and inferior directions from the sagittal and coronal plane views was 3.57 ± 0.03 mm.

**Table 1:** Measured parameters and values set based on recommendations and intended practices

Penumbral width	3.57 mm	3.57 mm
Systematic error	0.611 mm	0.611 mm
Random error	1.31 mm	1.31 mm
Fractions	5	1
Population percentage	90%	90%
Dose percentage	90%	90%
Resulting margin	2.42 mm	3.61 mm

These values were input into the margin recipe and will be used as the standard values with which to compare for sensitivity testing

The margins calculated with the SDE2 algorithm for the clinical parameters chosen and tabulated in Table 1 were 2.42 and 3.61 mm respectively for five fractions and one fraction.

**Fig. 3:**
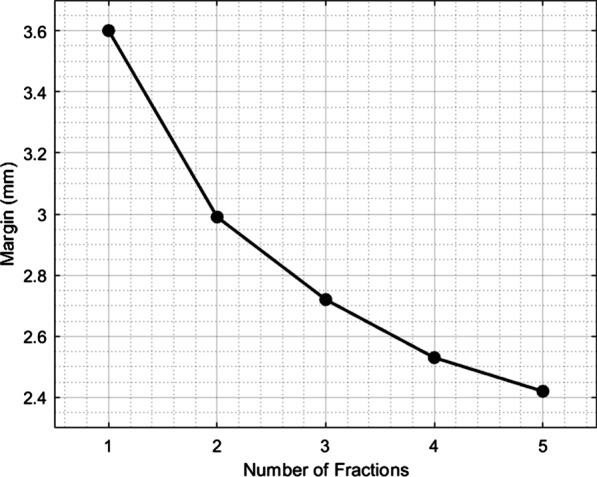
Results of sensitivity testing for margin dependence on number of fractions

**Fig. 4:**
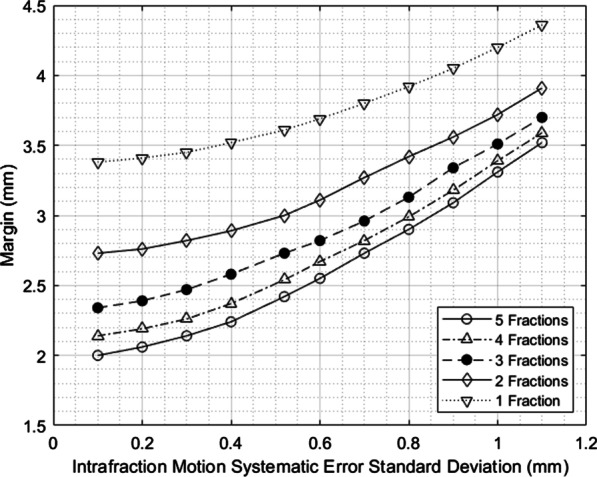
Sensitivity testing for margin dependence on random error magnitude over various numbers of fractions

**Fig. 5:**
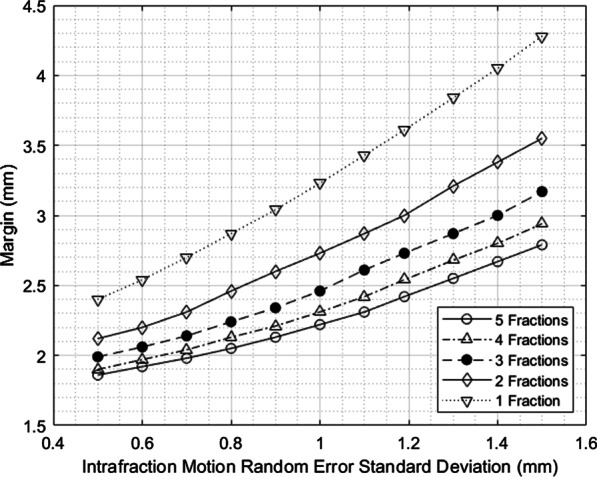
Sensitivity testing for margin dependence on systematic error magnitude over various fraction numbers

The margin values reported in Figs. 3, 4 and 5 show the sensitivity of the margin to selected input parameters to the margin calculation: fraction number and the random and systematic components of error standard deviation.

The results of the margin verification and testing are tabulated in Table 2. The investigated margins were: the analytical margin applied isotropically, the analytical margin applied in the superior and inferior directions only, and the commonly used 2 mm margin. The original PTV with no margin added was also investigated. Table 2 shows that the percentage of the population passing the dose and spinal cord criteria was closest to its intended value of 90% for the analytical margin when applied in the superior/inferior direction only.

**Table 2:** Results of margin validation

	Five-fraction plan				Single-fraction plan
	No margin (%)	Current margin 2 mm isotropic (%)	Analytical margin 2.4 mm isotropic (%)	Analytical margin 2.4 mm SI only (%)	Analytical margin 3.6 mm SI only (%)
> Rx dose to > 90% of CTV	81	100	100	96	95
< Max tolerance dose to cord	100	100	100	100	99
< Tolerance dose to < 10% of cord	99	99	99	100	95
Passing all criteria	81	99	99	96	93

Percentages of 140 patient population passing the required DVH criteria for a spinal SBRT plan with ± 5% error

**Fig. 6:**
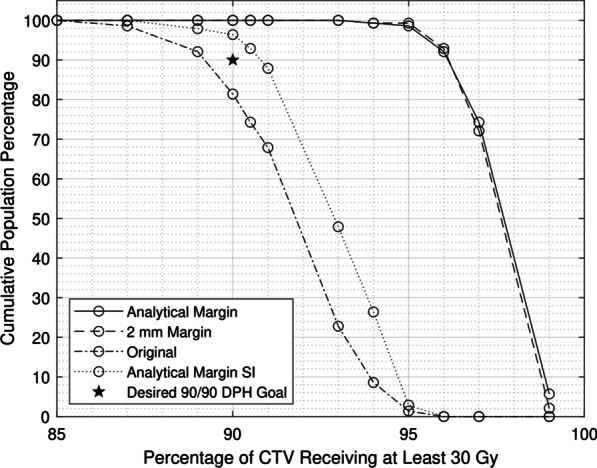
Dose population histograms resulting from the addition of different margins

Figure 6 shows the DPHs resulting from the addition of the three different margins and the original plan with no margin added for the five-fraction case. The desired treatment outcomes are summarized by the “Desired 90/90” blue point. The DPH for the plan created with the analytical margin applied in the superior/inferior direction only shows that it came the closest to achieving greater than 90% CTV coverage for 90% of patients. The DPH for the original plan shows that it failed to achieve the DPH goal and did not achieve coverage for enough patients. Only about 80% of patients received at least the full prescription dose to at least 90% of the CTV. Also, with no margin added, it is expected that the organ at risk constraints to the cord would not be significantly affected, and only one patient did not meet the constraint of less than 23 Gy to 10% of the cord. Both plans with the isotropic margins added far exceeded the DPH goal, meaning more normal tissue would be irradiated than is necessary to meet the stated treatment objectives. With the analytical margin added in all directions, again only one patient did not meet the cord criteria, but 100% of the 140 simulated patients received greater than 90% coverage of the CTV with the prescription dose. Even with the 5% uncertainty, approximately 95% of patients still would have received enough dose to enough of the CTV for both fractionation schemes. All plans shown achieved at least 85% coverage of the CTV for all patients.

## Discussion

The margins calculated in this work using the SDE2 algorithm are conservatively appropriate for use at our institution with the proposed spinal SBRT treatment protocol. The use of a PTV margin derived from known errors ensures sufficient coverage of the target volume while minimizing the dose to surrounding normal tissue. This involves using vacuum bag immobilization for a dual-arc 6 FFF VMAT treatment, using the same workflows and protocols for patient setup using CBCT images, and the same equipment and QA procedures to ensure the end-to-end system accuracy is still representative of the treatment delivery system. However, a user at another institution may measure these parameters and utilize the SDE2 calculation in the same way to generate an appropriate margin.

The margin formulation shows different sensitivities to the various input parameters, so uncertainties in these parameters could influence the accuracy of the margin calculated. The random and systematic intrafraction error standard deviations are the largest error components and the most likely to change because in this work, actual spinal SBRT patients were not being treated yet and could not be measured, so the true values may differ slightly. Also, the immobilization device used could be changed more easily than other treatment components like the linear accelerator. The margin is sensitive to the intrafraction motion error components with a nearly linear relationship for a five-fraction treatment in the region about the measured errors.

Based on the results of computing the dose received to the CTV and the spinal cord for simulated patients, a 2.4 mm margin applied only in the superior/inferior direction was sufficient to guarantee at least 90% of the prescription dose for at least 90% of simulated patients. The cord dose was less than the tolerance values for 100% of patients with this margin added. However, this formulation does not account for differences in importance between different areas of the CTV to receive the prescription dose. This formulation can only guarantee that some 90% of the volume will receive the prescription dose, but the particular volume may be variable. However, the results in Table 2 show that with the errors measured in this work, even the calculated margin of 2.42 mm applied in all directions did not cause the cord dose to exceed tolerances for 99% of cases. A margin providing the desired coverage level of the CTV which does not expand the treated volume towards the spinal cord is preferable, since moving the treated area closer to this critical structure increases the likelihood of exceeding the dose tolerances for the cord in the presence of uncertainties.

The measured percentage of the population meeting the CTV dose criteria was larger than what the margin calculation guaranteed likely due to some of the conservative assumptions made in the margin formulation. One conservative assumption was the perfect conformity of the prescription dose to the target [2]. The margin formulation assumes that with any shift, there is a loss of dose and coverage to the CTV. But in this work the CTV had an irregular shape, the spinal cord was in close proximity, and the minimum requirement for coverage of the PTV was 90%. When errors were introduced, the coverage did not necessarily decrease as fast as the margin recipe assumed or even decrease at all. Another assumption was that the minimum penumbra used in the calculation described the Gaussian penumbra of the dose-fall off for the entire target. But if a shift occurred in a direction with a wider penumbra, less dose was missed to the CTV than if the direction of the shift were along the minimum penumbra direction. The irregular shape also means the dose fall-off may not follow a Gaussian shape and may not decrease as quickly. The SDE2 algorithm generates a margin applied as an isotropic, spherical expansion, which may explain why the measured target coverage was higher than expected with the isotropic margin expansion. This led to the testing of the cylindrical margin with only superior and inferior expansion with a magnitude generated from the SDE2 algorithm as if it were to be applied isotropically. Although the SDE2 algorithm does not calculate a cylindrical margin, the superior/inferior margin results more closely aligned with the predicted criteria of 90% coverage for 90% of patients, likely due to the penumbra in this direction matching the algorithm input parameter, and the plan having a wider penumbra in all other directions. The algorithm was also designed to guarantee that the minimum dose point on the CTV does not fall below a desired level, which results in a higher dose to the entire volume.

One limitation of this work is the lack of error measurements on spinal SBRT patients treated at our institution, especially for the intrafraction motion measurements. However, the pelvis patients measured had similar treatment parameters. An area of future work would be to measure the intrafraction motion of these patients and compare those results to those of this work for a potentially different margin result. Another limitation is that only one type of plan with one particular shape for the CTV was used for validating the margin. There are many different shapes the CTV can take based on the extent of the disease and its invasion to other areas of the vertebral bodies. This work used a plan with a T-spine lesion, which is the most common region for secondary metastasis [1]. Future work could include validating the margin for different plans with different CTV shapes, potentially where the CTV would include the spinal cord. Other types of treatment geometries may be used to treat spinal SBRT, such as using couch kicks and non-coplanar arcs, and the results from this work may not apply to those types of treatments. The minimum penumbral width may occur in a different direction other than superior/inferior which would impact how the margin works to ensure coverage of the CTV. It may also impact the spinal cord dose if the dose distribution has a significantly different shape in that region. Another limitation was that different plans had to be created for each new volume created by the addition of a new margin. Efforts were made to ensure similarity between these plans, but in order to achieve the fairest comparison, the plans should have nearly identical dose distributions inside the CTV and outside of it to avoid any bias of one plan having a more favorable dose distribution from the start.

## Conclusions

This work showed that 96 ± 5% of patients would have sufficient target coverage with the prescription dose with a 2.42 mm SI margin applied, but this does not imply that the remaining percentage of patients would necessarily fail treatment. The results of this work support the hypothesis that a margin can be determined for spinal SBRT which meets the treatment goals of greater than 90% of the prescription dose to greater than 90% of the population without exceeding the cord dose tolerances. The treatment goals were conservatively met with a 2.42 mm SI margin for five fractions and a 3.60 mm SI margin for a single-fraction dual-arc VMAT spinal SBRT treatment with vacuum bag immobilization at Mary Bird Perkins Cancer Center. All sources of known measurable error during radiotherapy were included in the margin formulation and the margin was verified on a clinical spinal SBRT treatment plan.

## Data Availability

The datasets used and/or analyzed during the current study are available from the corresponding author on reasonable request.
